# Uromodulin deficiency alters tubular injury and interstitial inflammation but not fibrosis in experimental obstructive nephropathy

**DOI:** 10.14814/phy2.13654

**Published:** 2018-03-29

**Authors:** Olena Maydan, Paul G. McDade, Yan Liu, Xue‐Ru Wu, Douglas G. Matsell, Allison A. Eddy

**Affiliations:** ^1^ Department of Pediatrics University of British Columbia and British Columbia Children's Hospital Research Institute Vancouver British Columbia Canada; ^2^ Department of Urology New York University New York New York

**Keywords:** Chronic kidney disease, kidney injury molecule‐1, uromodulin

## Abstract

Human GWAS and Mendelian genetic studies have linked polymorphic variants and mutations in the human uromodulin gene (*UMOD*) with chronic kidney disease. The primary function of this kidney‐specific and secreted protein remains elusive. This study investigated whether UMOD deficiency modified responses to unilateral ureteral obstruction (UUO)‐induced kidney injury. Kidneys harvested from groups of wild‐type (UMOD+/+) and knockout (UMOD−/−) male mice (*n* = 7–10 each) were studied on days 7, 14, and 21. Compared to sham kidneys, UMOD protein levels increased 9–13x after UUO and were associated with increased urinary UMOD levels. Kidney KIM‐1 protein levels were higher in the UMOD−/− groups at all time‐points (4–14x). The UMOD−/− groups also had higher KIM‐1 kidney‐to‐urine relative ratios (5–35x). In vitro studies using KIM‐1 expressing 769‐P cells showed lower KIM‐1 levels in the presence of UMOD protein. Levels of proapoptotic genes and the epithelial cell apoptotic protein marker M30 were significantly lower in the UMOD−/− groups. Both M30 and KIM‐1 colocalized with intraluminal UMOD protein deposits. Interstitial inflammation was less intense in the UMOD−/− groups. Renal fibrosis severity (kidney collagen mRNA and protein) was similar in both genotypic groups on days 7, 14, and 21. Our findings suggest a role for UMOD‐dependent inhibition of KIM‐1 expression and its apoptotic cell scavenging responses during chronic obstruction‐associated tubular injury.

## Introduction

Uromodulin (UMOD), also known as Tamm‐Horsfall protein (Pennica et al. 1987), is the most abundant protein in normal urine. Despite its discovery more than 60 years ago, the primary physiological function of UMOD is still not completely understood. UMOD is a kidney‐specific 85–95 kDa glycoprotein uniquely produced by the thick ascending limb of the loop of Henle (TALH) and the early distal tubule. It traffics to the apical membrane where it is anchored by glycophosphatidylinositol until it is cleaved by an unknown protease and shed into the luminal space. The following functions have been proposed for UMOD: (1) Bladder mucosal defense against urinary tract infections (Bates et al. 2004; Saemann et al. 2005a); (2) An endogenous inhibitor of stone formation (Liu et al. 2010; Wolf et al. 2013; Iorember and Vehaskari 2014); (3) Regulation of renal sodium and potassium transport and blood pressure control (Mutig et al. 2011; Renigunta et al. 2011; Trudu et al. 2013); (4) Regulation of innate immune responses (Saemann et al. 2005b; Rhodes 2006; Rampoldi et al. 2011; Darisipudi et al. 2012; Liu et al. 2016); (5) A ligand for the scavenger receptors (Pfistershammer et al. 2008); (6) Expressed and perhaps functionally active in the apical primary cilia of epithelial cells of the TALH (Zaucke et al. 2010).

Renewed interest in deciphering the role of UMOD in the kidney has been catalyzed by two important observations in humans. First, patients expressing a mutant UMOD protein as a result of an autosomal dominant mutation develop chronic tubulointerstitial disease (ADTKD) (Eckardt et al. 2015) that typically progresses to end‐stage kidney disease between the fourth and seventh decade of life (Rampoldi et al. 2011; El‐Achkar and Wu 2012; Iorember and Vehaskari 2014)( Rampoldi et al. 2003; Bernascone et al. 2006; Schaeffer et al. 2009). Second, several large and independent genome‐wide association studies (GWAS) have reported a strong association between specific polymorphic variants in the promoter region of the *UMOD* gene and the risk and/or severity of chronic kidney disease (CKD) (Kottgen et al. 2009, 2010; Chambers et al. 2010; Boger et al. 2011; Liu et al. 2011; Rampoldi et al. 2011; Pattaro et al. 2012, 2016; Reznichenko et al. 2012; Olden et al. 2013; Trudu et al. 2013; Devuyst and Bochud 2015). Subsequent studies have queried whether urinary UMOD levels could be used as a reliable marker to predict CKD progression rates, with conflicting results (Sejdiu and Torffvit 2008; Han et al. 2013; O'Seaghdha et al. 2013; Olden et al. 2014; Devuyst and Bochud 2015; Garimella et al. 2015); overall the data suggest that the urinary UMOD level is a marker of the functional nephron mass. Recognizing that urine sample processing and handling substantially influences urinary UMOD levels (Youhanna et al. 2014), recent human studies have focused on serum UMOD levels, with evidence that lower levels are associated with lower GFR, increased cardiac mortality and impaired glucose metabolism (Delgado et al. 2017; Kraus and Wanner 2017; Leiherer et al. 2017).

Much less is known about UMOD expression and function during kidney injury. The kidneys of mice with genetic UMOD deficiency develop normally and do not develop spontaneous tubulointerstitial disease (Raffi et al. 2006). These mice are more susceptible to urinary tract infections (Bates et al. 2004) and with advanced age many of the mice have evidence of renal calcifications (Liu et al. 2010). In a mouse model of ischemia‐reperfusion injury, kidney UMOD expression was biphasic – declining during the acute injury phase followed by significant upregulation during recovery. In this model, UMOD expression has been shown to serve a protective role, as tubular necrosis severity was significantly worse in UMOD‐deficient mice (El‐Achkar et al. 2008, 2013). Given its reported pleiotropic effects, it has been argued that structurally normal UMOD could serve either harmful or beneficial roles in chronic kidney disease (CKD) (Eddy 2011). These possibilities have not yet been investigated in animal models of progressive kidney injury.

This study was designed to investigate the hypothesis that UMOD serves a functionally significant and nonredundant role in the inflammatory and fibrotic responses that typify CKD and to elucidate potential mechanisms. The well‐characterized mouse model of unilateral obstruction (UUO) was selected (Eddy et al. 2012), reasoning that this model should enhance intraluminal UMOD accumulation and maximize the potential to discern response differences between normal and UMOD‐deficient mice. This study reports that the absence of UMOD led to distinct differences in the cellular response to injury but did not impact overall kidney fibrosis severity.

## Materials and Methods

### Animal models

UMOD−/− mice were generated on a 129SvEv background using mice purchased from Taconic Biosciences (Albany, NY, USA) by Mo et al. (2004). Breeding pairs of mice were obtained from Dr. Terek M. El‐Achkar (IU Health University Hospital Indianapolis, USA) and were bred in our animal facility for this study. The *UMOD* genotype of all mice was confirmed by PCR. Unilateral ureteral obstruction (UUO) was performed as described previously (Oda et al. 2001) using 10‐ to 12‐week‐old males in all groups. Groups of UMOD+/+ and UMOD −/− mice (*n* = 10/group at each time‐point) were sacrificed 7, 14, and 21 days after sham and UUO surgery. Kidneys from UUO and sham animals were harvested and cut in an identical fashion into sections for the total collagen assay, protein and RNA isolation, and histological assessment and processed as described by Kim et al. (2001).

All animal breeding and experimental procedures were approved by the University of British Columbia Animal Care Committee.

### Antibodies

Primary antibodies used in the study were: sheep‐anti mouse UMOD, goat‐anti‐mouse KIM‐1, mouse‐anti‐human KIM‐1, goat‐anti mouse NGAL (R&D Systems Inc, Minneapolis, MN, USA); sheep‐anti‐human UMOD, rat anti‐mouse F4/80 (AbD Serotec/Bio‐Rad, Mississauga, ON, Canada); mouse anti‐mouse *α*SMA, rabbit anti‐human fibronectin (Sigma‐Aldrich, St. Louis, MO, USA); rabbit anti‐mouse/human/rat TRPV5 (Alomone Labs, Jerusalem, Israel); mouse anti‐human PCNA (eBioscience/Thermo Fisher Scientific, Waltham, MA, USA); rabbit anti‐mouse/human/monkey megalin/LRP‐2 (Abcam, Toronto, ON, Canada); rabbit‐anti‐mouse Laminin (Millipore/Sigma), mouse‐anti‐mammalian acetylated *α*‐Tubulin (Santa Cruz Biotechnology, Dallas, TX, USA), and rabbit anti‐mouse GAPDH (Cell Signaling Technology, Danvers, MA, USA).

Secondary horseradish peroxidase (HRP) conjugated anti‐IgG antibodies were: donkey anti‐sheep (Jackson ImmunoResearch Labs, West Grove, PA, USA); goat anti‐rat and horse anti‐mouse (Cell Signaling Technology); goat anti‐rabbit and donkey anti‐goat (Santa Cruz Biotechnology).

Secondary antibodies used in immunofluorescent microscopy were highly cross‐absorbed Alexa Fluor 488 or 568 conjugated antibodies (Invitrogen/ThermoFisher Scientific) for detection of mouse, rabbit, rat, sheep, and goat IgG.

### Cell lines and transfection experiments

Human renal adenocarcinoma cells 769‐P (CRL‐1933) purchased from ATCC (Manassas, VA, USA) were grown in RPMI 1640 Medium supplemented with 10% FBS (Gibco/Thermo Fisher Scientific, Waltham, MA, USA). Human embryonic kidney 293 cells (HEK‐293) purchased from Sigma‐Aldrich (St. Louis, MO, USA) were grown in MEM Medium supplemented with 2 mmol/L glutamine, 1% nonessential amino acids and 10%FBS (Gibco/Thermo Fisher Scientific). Cells were transfected with human UMOD construct expressed in the pcDNA3.1 vector (gift from Dr. L. Rampoldi, Dulbecco Telethon Institute, Milan, Italy) (Bernascone et al. 2006) using jetPRIME transfection agent (Polyplus Transfection, Illkirch, France), according to the manufacturer's instructions. For in vitro KIM‐1 expression experiments, 769‐P and 293 cells transfected with pcDNA3.1 vector alone (Control, pcDNA3.1‐C) and full length UMOD in pcDNA3.1 vector (pcDNA3.1‐UMOD) were reseeded 4 h after transfection into cell culture inserts with 1.0 *μ*m pores (BD Biosciences, Mississauga, ON, Canada). Twenty‐four hours after transfection, the inserts were transferred to six‐well plate containing 293‐C and 293‐UMOD transfected cells, and incubated for an additional 24 h. Cells and conditioned medium were collected and analyzed for mRNA and protein levels of KIM‐1.

### mRNA levels

RNA was extracted using RNeasy Mini‐Kit (Qiagen, Valencia, CA, USA) using the manufacturer's instructions. Reverse transcription of mRNA was performed using the High Capacity RNA‐to‐cDNA kit (Applied Biosystems/Thermo Fisher Scientific, Waltham, MA, USA). Gene expression was determined by real‐time PCR using TaqMan probes and Applied Biosystems StepOnePlus machine (Applied Biosystems/Thermo Fisher Scientific). PCR product levels were normalized to GAPDH levels in the same sample. Results for each time‐point were expressed relative to the UMOD+/+ sham data. The Applied Biosciences TaqMan Probe numbers were:

Collagen 1*α*1 Mm00801666_g1; Collagen 3*α*1 Mm01254476_m1;

UMOD Mm00447649_m1; TRPV5 Mm01166037_m1; NKCC2 Mm01275821_m1;

ROMK2 Mm00444727_s1; MCP‐1 Mm00441242_m1; KIM‐1 Mm00506686_m1; FASL Mm00438864_m1, TNF*α* Mm00443285; mouse GAPDH Endogenous Control Mm99999915_g1

### Western blot analysis

Protein was extracted from kidneys as described by Hiatt et al. (2013). Briefly, flash frozen kidney tissue samples were homogenized with BioPulverizer (Biospec Products, Bartlesville, OK, USA) and lysed in buffer containing T‐PER Tissue Protein Extraction Reagent (Thermo Fisher Scientific) and SDS Laemmli buffer in 1:1 ratio. Cells were lysed in RIPA buffer containing 50 mmol/L Tris (pH 7.5), 150 mmol/L NaCl, 1% deoxycholic acid, 1% NP40, 0.1% SDS. Both lysis buffers were supplemented with Complete Protease Inhibitors Cocktail tablets (Roche, Laval, QC, Canada). Protein concentration was measured using a bicinchoninic acid protein assay kit (Pierce/Thermo Fisher Scientific) and equal amounts of total protein were loaded into 6% or 8% polyacrylamide gels for SDS‐PAGE electrophoresis. Proteins were transferred to polyvinylidene difluoride membrane (Millipore, Etobicoke, ON, Canada). Blots were blocked with 5% fat‐free milk (Cell Signaling Technology) in TBS‐T (50 mmol/L Tris, 150 mmol/L NaCl, 0.1% Tween‐20, pH 7.4) and incubated with primary antibodies at 4°C overnight. Blots were incubated with horseradish peroxidase–labeled secondary antibody for 1 h at 24°C and then developed with Clarity Western ECL detection system (Bio‐Rad, Mississauga, ON, Canada). Visualization was done with ChemiDoc XRS+ and Quantity One Software version 4.6.9 (Bio‐Rad). Blots were subsequently stripped and reprobed with anti‐GAPDH antibodies (Cell Signaling Technology) as the protein loading control. Quantification was done using Image Lab software version 4.0 (Bio‐Rad). Results for each time‐point were expressed relative to the UMOD+/+ sham data.

### Urinary UMOD and KIM‐1 Levels

For UMOD detection, urine was collected from the dilated renal pelvis of the UUO mice and the bladders of sham animals. Samples were diluted in Triton‐EDTA (TEA) buffer (0.5% Triton X‐100, 20 mmol/L EDTA pH 7.5) supplemented with Complete Protease Inhibitor Cocktail tablets for UMOD (Lau et al. 2008) and in water for creatinine measurements. To evaluate the amount of insoluble UMOD, after overnight storage at 4°C, diluted urine samples were vortexed and centrifuged as described by Thomas et al. (2010). Precipitates were dissolved in SDS Laemmli buffer in a volume equal to the volume of diluted urine sample. UMOD levels were detected by western blotting, as described previously, using equal volumes of supernatants (soluble UMOD) and dissolved pellets. Results of densitometry analyses for soluble and insoluble UMOD in each urine sample (*n* = 4 for UUO group and *n* = 3 for sham group) were normalized to creatinine levels in the same sample. Creatinine levels were determined using Creatinine Parameter Assay Kit (R&D Systems). KIM‐1 was measured in urine samples stored at −80°C. Urine samples from sham animals (*n* = 4) and UUO mice (*n* = 7) were briefly vortexed and 5 *μ*L was added to equal volumes of SDS Laemmli buffer. Samples were analyzed by western blotting procedure, and results of densitometry for KIM‐1 were normalized to creatinine levels, as described for UMOD.

### Immunohistochemical staining and microscopy

Staining was performed on sections of formalin fixed and paraffin embedded kidneys.

Sections were processed as described by Hiatt et al. (2013). Secondary antibody used were Alexa‐Fluor conjugated (Molecular Probes/ThermoFisher Scientific). Slides were mounted in ProLong Gold Antifade Mountant with DAPI (4′,6‐diamidino‐2‐phenylindole) (Invitrogen/ThermoFisher Scientific). Standard negative controls for all staining procedures included staining with secondary antibody alone. All slides were coded to ensure that the observer was blinded to the animal group at the time of quantitative analyses. Epifluorescence microscopy was performed using a Leica DM4000B microscope. Images were captured and processed using OpenLab software (Perkin Elmer, Waltham, MA, USA). Confocal analysis was performed using a Leica SP5 II Confocal microscope with Leica's LAS AF software for image acquisition.

### Apoptosis

The degree of renal epithelial cell apoptosis was evaluated by measuring caspase‐cleaved cytokeratin 18 protein fragment (M30) levels in kidney tissue sections (Caulin et al. 1997). M30 was detected using mouse M30 CytoDeath antibody (Roche) followed by anti‐mouse Alexa Fluor 488 secondary antibody (Molecular Probes/ThermoFisher Scientific). Samples were examined by epifluorecsence microscopy, and positive M30 staining area was calculated using Image J software (National Institutes of Health, Bethesda, MD, USA). For each sample, results were expressed as the average positive area from 10 random fields of view at ×40 magnification.

### Kidney total collagen levels

Total collagen was determined using the hydroxyproline assay as described previously (Matsuo et al. 2005). For quantification of the collagen positive interstitial area, tissue sections were stained with picrosirius red and analyzed by polarized light on DX61 microscope using cellSens image acquisition/analysis software (Olympus, Center Valley, PA, USA). For each sample 10 random 40x magnification images were captured and the mean positive area was calculated using ImageJ software (NIH).

### Statistical analysis

Results are calculated as mean ± 1 SEM levels. Sample sizes were as follows: UUO group *n* = 7 and sham group *n* = 5 for the protein expression experiments and UUO group *n* = 5 and sham group *n* = 3 for the mRNA experiments. Total collagen assay was performed with 10 animals in all UUO and sham groups. For the image analysis data, the mean for each group was calculated from individual results of 5–7 animals in UUO groups, and three animals in sham groups. Statistical significance between two experimental groups was evaluated using the unpaired *t* test for parametric data using PRISM 7.0a software (GraphPad, San Diego, CA, USA). Statistical significance of kidney UMOD mRNA and protein levels relative to sham levels were analyzed by one‐way ANOVA with Bonferoni's multiple comparison test. Differences with *P *<* *0.05 were considered statistically significant.

### Bacterial detection in tissue sections

The kidneys of UUO group animals were analyzed for the presence of bacteria by microscopic examination of tissue sections stained using the modified Brown‐Hopps method that detects both gram‐positive and gram‐negative bacteria on formalin‐fixed paraffin embedded samples (Luna 1968). The staining kit was from Electron Microscopy Sciences (Hatfield, PA, USA), and sections were processed according to manufacturer's instruction. Sections were inspected by two independent observers under ×100 magnification using a Leica DM4000B microscope. Positive controls for this experiment were sections of mouse colon from animals with induced colitis and inflammation (gift of Dr. Laura Sly (UBC, Vancouver, Canada). The negative controls were kidney sections from 4‐week‐old control mice (not sham).

### Kidney calcium deposition

For visualization of phosphate and calcium deposits in the kidneys of UUO mice, formalin‐fixed paraffin‐embedded tissue section were stained with Von Kossa and Alizarin Red stains, respectively. All staining reagents were from Sigma‐Aldrich. Briefly, for Von Kossa staining sections were deparaffinized, hydrated, and incubated in 2% aqueous silver nitrate solution under direct bright light for 1 h. After washing for 5 min in 5% sodium thiosulfate, sections were counterstained with 0.1% Nuclear Fast Red solution. For Alizarin Red staining, deparaffinized and hydrated sections were treated with 2% Aqueous Alizarin Red solution for 2 min. Following both protocols, sections were dehydrated, mounted, and inspected under light microscope (Leica DM4000B) by two independent observers. Positive controls were sections of embryonic (E17.5) mouse limbs and negative controls were kidney sections from UMOD+/+ 4‐week‐old mice.

## Results

### UMOD expression

**Figure 1:**
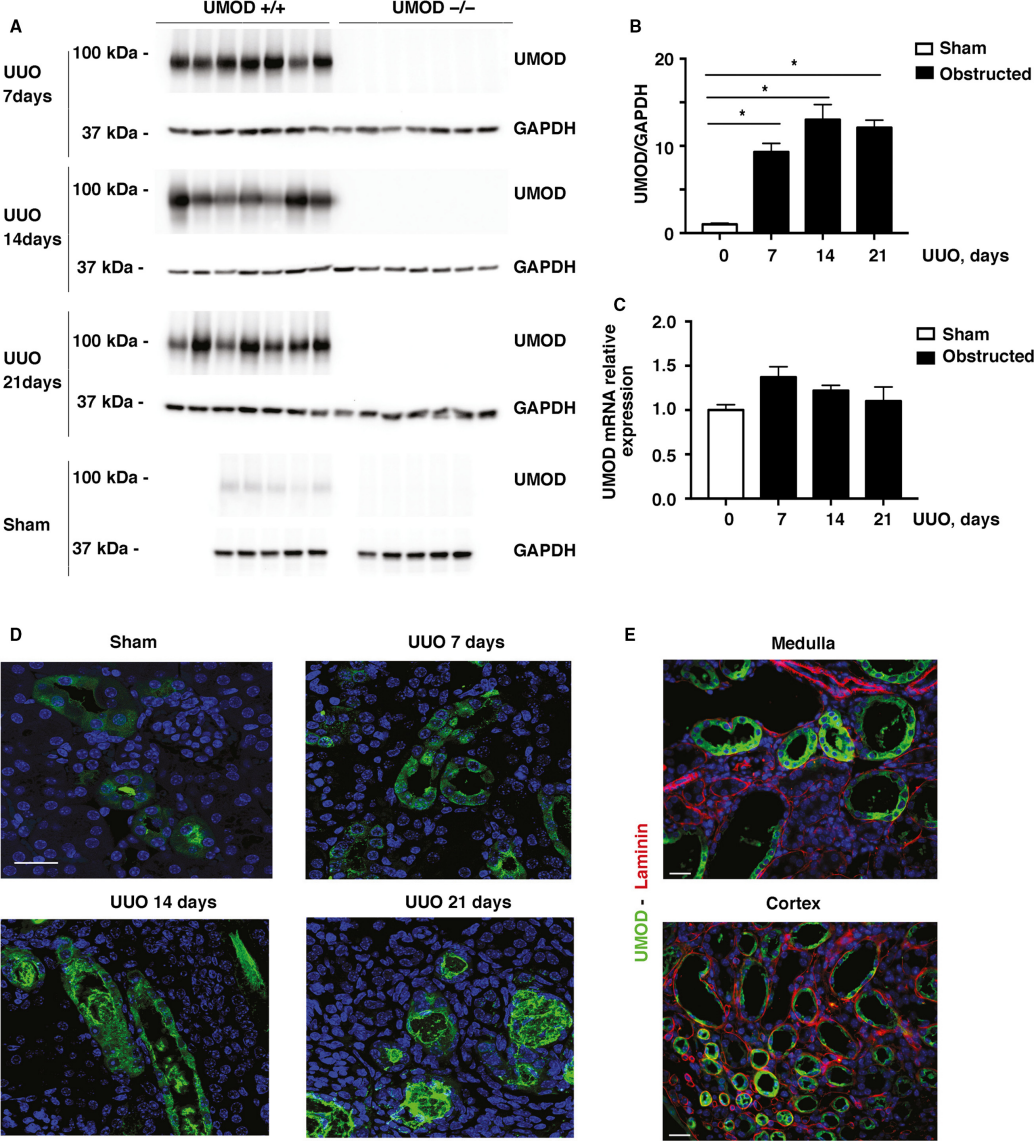
Kidney UMOD protein levels increase in UUO‐induced injury. Total kidney UMOD protein levels increased significantly 7, 14, and 21 days after UUO (*n* = 7/group) compared with the sham group (*n* = 5/group) in the UMOD+/+ mice, as measured by western blotting (A). UMOD protein was not detected in any of the UMOD‐/‐ mice. Mean levels + 1SEM shown in bar graphs were calculated after loading correction using GAPDH levels (B). * indicates a *P* value < 0.05 relative to sham. Kidney *Umod* mRNA levels measured by real‐time PCR were not significantly different from sham levels at any time‐point after UUO (C). Representative confocal immunofluorescence photomicrographs of the UMOD+/+ kidneys illustrate UMOD protein within tubular epithelia and their adjacent lumina (D). By days 14 and 21, intraluminal deposits are predominant and acquire an organized structural appearance by day 21. Costaining for laminin (red) to delineate tubular basement membranes failed to detect convincing interstitial UMOD deposits (green) by epifluorescence microscopy, as shown on day 14 (E). Magnification: ×100 for confocal and ×40 for nonconfocal microscopy. Scale bars: 25 μm.

Kidney UMOD protein levels were increased in response to UUO. By western blotting, total kidney UMOD levels were increased 9‐13 fold compared to the levels in sham kidneys on days 7, 14, and 21 post UUO surgery, respectively (Fig. 1A and B). As expected, no UMOD protein was detected in any of the UMOD−/− mice (data not shown). Kidney *Umod* mRNA levels measured by qPCR were not significantly higher in the UUO UMOD +/+ mice compared to the sham group (Fig. 1C), suggesting that UMOD retention within obstructed kidneys was the primary mechanism for the observed increases in UMOD protein.

By immunofluorescence microscopy, UMOD was expressed on the apical membrane of subsets of tubules in the cortex and medulla, reflecting its known expression by TALH and early distal convoluted tubules in normal kidneys (Fig. 1D). Following UUO, the tubular distribution of UMOD was similar, but staining intensity was increased, both intracellular and within tubular lumina. There was evidence of retrograde movement of intraluminal UMOD, even appearing in Bowman's space of some glomeruli. At later time‐points (days 14 and 21) the intratubular deposits often acquired a complex organized structure. Using UMOD and laminin dual staining to delineate tubular basement membranes, UMOD was not detected within interstitial spaces (Fig. 1E).

**Figure 2:**
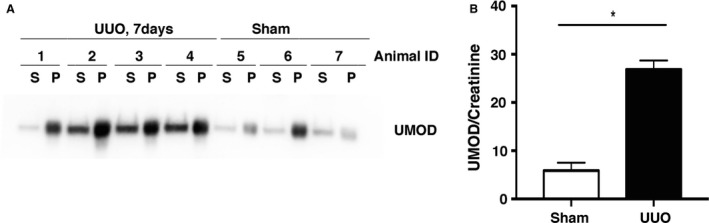
Urinary UMOD levels are increased in UUO nephropathy (day 7). Since UMOD protein rapidly precipitates in urine samples, the extent of which varies with storage conditions, these data are based on results from the analysis of fresh urine samples aspirated from the dilated renal pelvis (UUO mice) or bladders (sham mice) of the UMOD+/+ mice. Samples were diluted in TEA buffer. After overnight storage at 4°C, samples were centrifuged to precipitate the insoluble fraction that was dissolved in SDS Laemmli buffer, in a volume equal to the volume of the diluted urine samples. After loading equal volumes, western blotting detected UMOD protein in both the soluble (S) and insoluble pellet (P) factions of the urine collected from the UMOD+/+ mice (A). Total urinary UMOD levels (soluble + pellet) (arbitrary densitometric units/ml) adjusted to urinary creatinine concentration (mg/mL) were significantly higher (*) in the UUO group (*n* = 3 sham and *n* = 4 UUO mice) (B). Results are means + 1SEM. * indicates a *P* value less than 0.05.

Measurement of urinary UMOD‐to‐creatinine ratios in samples of urine aspirated from the dilated renal pelvis of the UUO mice and from the bladders of the sham mice was complicated by the fact that the UMOD protein readily precipitated, both in fresh urine samples and to an even greater extent in samples stored at 4°C and −80°C (data not shown). For this reason, fresh urine samples were collected from a new group of day 7 UUO and sham mice (*n* = 3–4/group), immediately diluted in TEA buffer and analyzed after overnight storage at 4°C. Even with this rapid processing, some insoluble precipitates formed (Fig. 2A). Samples were centrifuged and the pellet solubilized in SDS buffer. Total urinary UMOD levels (soluble fraction plus the solubilized pellet fraction) corrected to urinary creatinine levels were 4.5 fold higher in UUO group compared to levels in sham mice (Fig. 2B).

### Tubular injury

**Figure 3:**
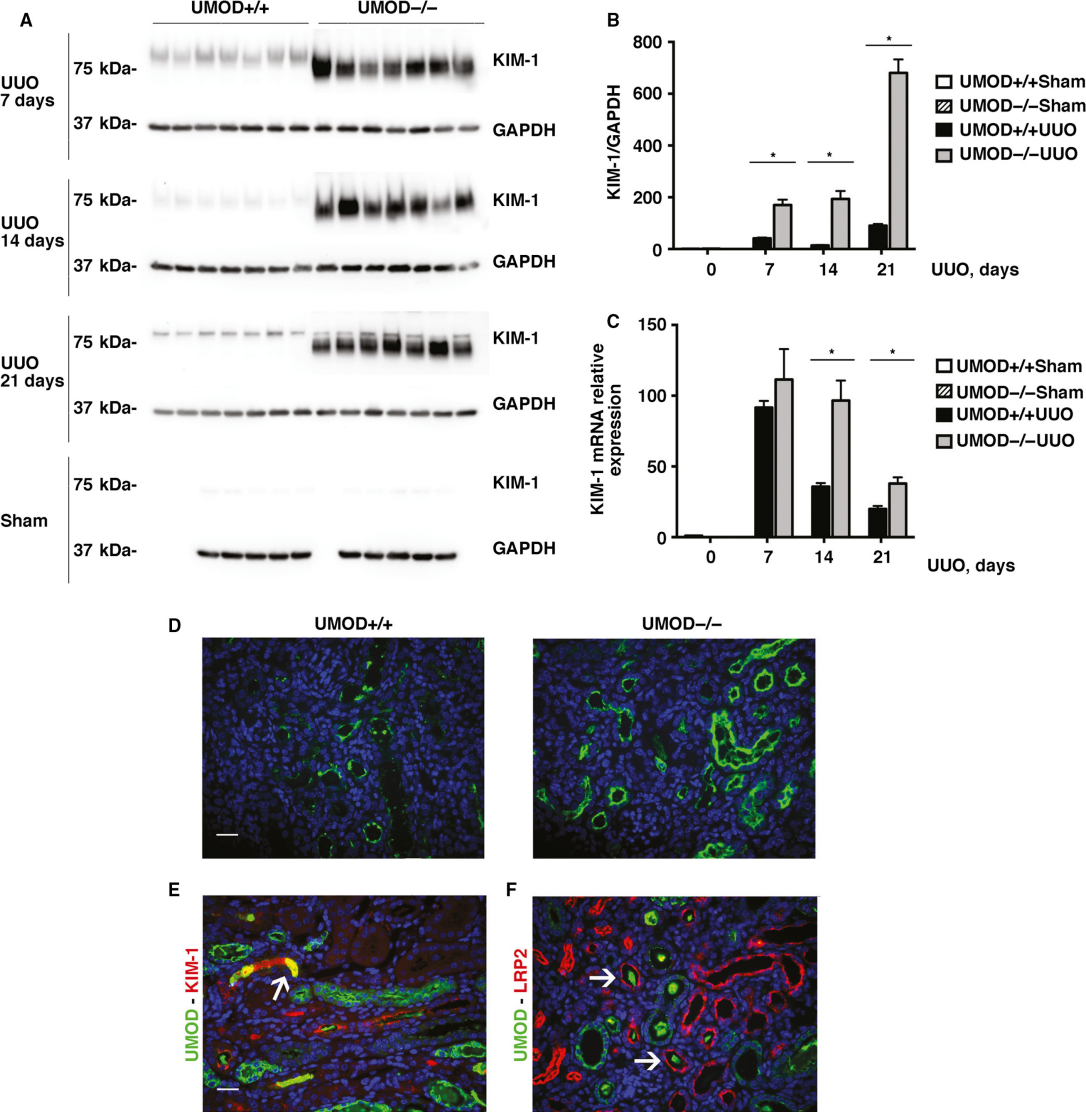
The tubular injury marker KIM‐1 is higher in mice lacking in UMOD during obstructive nephropathy. By western blotting, kidney KIM‐1 protein levels were increased after UUO (*n* = 5 for sham groups; *n* = 7 for UUO groups) at all time‐points (days 7, 14, and 21); the levels were significantly higher in the UMOD−/− groups. Results are corrected for protein loading using GAPDH levels. (A, B). Kidney KIM‐1 mRNA levels measured by real‐time PCR (*n* = 3 for sham groups; *n* = 5 for UUO groups) were significantly higher on UUO days 14 and 21 (C). Representative day 14 immunofluorescence photomicrographs illustrate more intense KIM‐1 staining in the UMOD−/− kidney (D). KIM‐1 staining was concentrated along the apical membrane with lesser amounts in tubular lumina; the latter more evident in the UMOD+/+ mice. Brightly stained ribbon‐like KIM‐1 +  bands, frequently observed along the apical membrane in the UMOD‐/‐ mice, were much less common in the UMOD+/+ mice. Dual staining for UMOD (green) and KIM‐1 (red) in a UMOD+/+ day 14 kidney illustrates an area of tubular intraluminal staining for both proteins (yellow; highlighted by the arrow) (E). Dual staining for UMOD (green) and the proximal tubular apical membrane protein LRP2 (red) in a UMOD+/+ day 14 kidney illustrates the presence of luminal UMOD deposits surrounded by LRP2 +  tubules (arrows) (F). Bar graphs represent means +1SEM; * indicates a *P* value < 0.05. Photomicrograph magnification: ×40. Scale bars: 25 μm.

Several markers of tubular injury and integrity were measured to determine if the absence of UMOD affected the ability of tubular epithelia to defend against obstruction‐induced kidney injury. Given the potential for cross‐talk between luminal UMOD and proximal tubules (Saemann et al. 2005b; Pfistershammer et al. 2008; Darisipudi et al. 2012; Mao et al. 2014), we first queried whether the expression of kidney injury molecule‐1 (KIM‐1) differed between the UMOD genotypes. Not detectable in normal kidneys, KIM‐1 is an epithelial phagocytic receptor that is uniquely expressed by damaged proximal tubular cells. In the UUO model, 90–100% of proximal tubules have been reported to express KIM‐1 (Humphreys et al. 2013). Beyond its proven value as tubular injury biomarker in numerous animal models and human kidney diseases, both acute and chronic (Yin et al. 2016), there is growing evidence that KIM‐1 serves a variety of functions that may either enhance or attenuate kidney injury, depending upon the context within which it is expressed (Humphreys et al. 2013; Yang et al. 2015; Yin et al. 2016). KIM‐1 protein levels were significantly up‐regulated in the UUO kidneys at all time‐points (days 7, 14, and 21), and KIM‐1 levels were significantly and remarkably higher in the UMOD−/− groups compared to the UMOD+/+ groups (4.3–13.7x) (Fig. 3A and B). Kidney *Kim‐1* mRNA levels measured by qPCR were significantly higher in the UMOD−/− on days 14 and 21 (Fig. 3C). By immunofluorescence staining, more positive tubules were observed in the UMOD−/− mice at all time‐points (Fig. 3D). KIM‐1 staining was concentrated along the apical membrane with lesser amounts in tubular lumina, with the latter more evident in the UMOD+/+ mice. Brightly stained ribbon‐like KIM‐1 +  bands, frequently observed along the apical membrane in the UMOD−/− mice, were not common in the UMOD+/+ mice. Dual staining for UMOD and KIM‐1 identified tubular deposits containing both of these proteins in the UMOD+/+ mice (Fig. 3E). The finding of UMOD protein deposits within the lumina of some megalin/low‐density lipoprotein‐related protein 2 [LRP2]‐positive tubules confirmed that some of the secreted UMOD protein moved ante‐grade as far as proximal tubules where KIM‐1 is produced after ureteral obstruction (Fig. 3F).

Urinary KIM‐1‐to‐creatine levels were higher in the UMOD+/+ groups, but levels were highly variable between mice (data not shown). This trend was remarkable given the opposite finding when kidney KIM‐1 proteins levels were measured, suggesting that UMOD influenced proximal tubular KIM‐1 retention. KIM‐1 kidney‐to‐urine relative protein ratios were 35x, 26x, and 5x higher in the UMOD−/− groups of days 7, 14, and 21, respectively (Table 1).

**Table 1:** KIM‐1 protein distribution pattern

KIM‐1 Kidney‐to‐Urine Relative Protein Ratios^a^				
	Sham	Day 7 UUO	Day 14 UUO	Day 21 UUO
UMOD+/+ groups	1.000	0.003	0.159	26.233
UMOD−/− groups	1.066	0.105	4.085	128.619
Kidney‐to‐urine relative ratios for UMOD−/− versus UMOD+/+ groups	1	35	26	5

a KIM‐1 protein levels in kidney tissue lysates were measured by western blotting and results were corrected for protein loading by measuring GAPDH levels. Final results were expressed in arbitrary units using the mean level in the UMOD+/+ sham group as 1.0 densitometric unit (a). KIM‐1 urinary levels were measured by western blotting and results were corrected to urinary creatinine levels measured in the same samples using the Creatinine Parameter Assay Kit. Final results were expressed in arbitrary units using the mean level in the sham UMOD+/+ group as 1.0 densitometric unit (b). Within each group, all protein or all urine samples (*n* = 7) were loaded into a single gel, as shown in Figure 3. The data shown is in the first and second rows are the ratios for the mean values of (a) divided by the mean value of (b) at each time‐point. The data in the final row summarizes the relatively higher expression of KIM‐1 protein in the kidney of the UMOD−/− mice (calculated data in second row ÷ first row).

**Figure 4:**
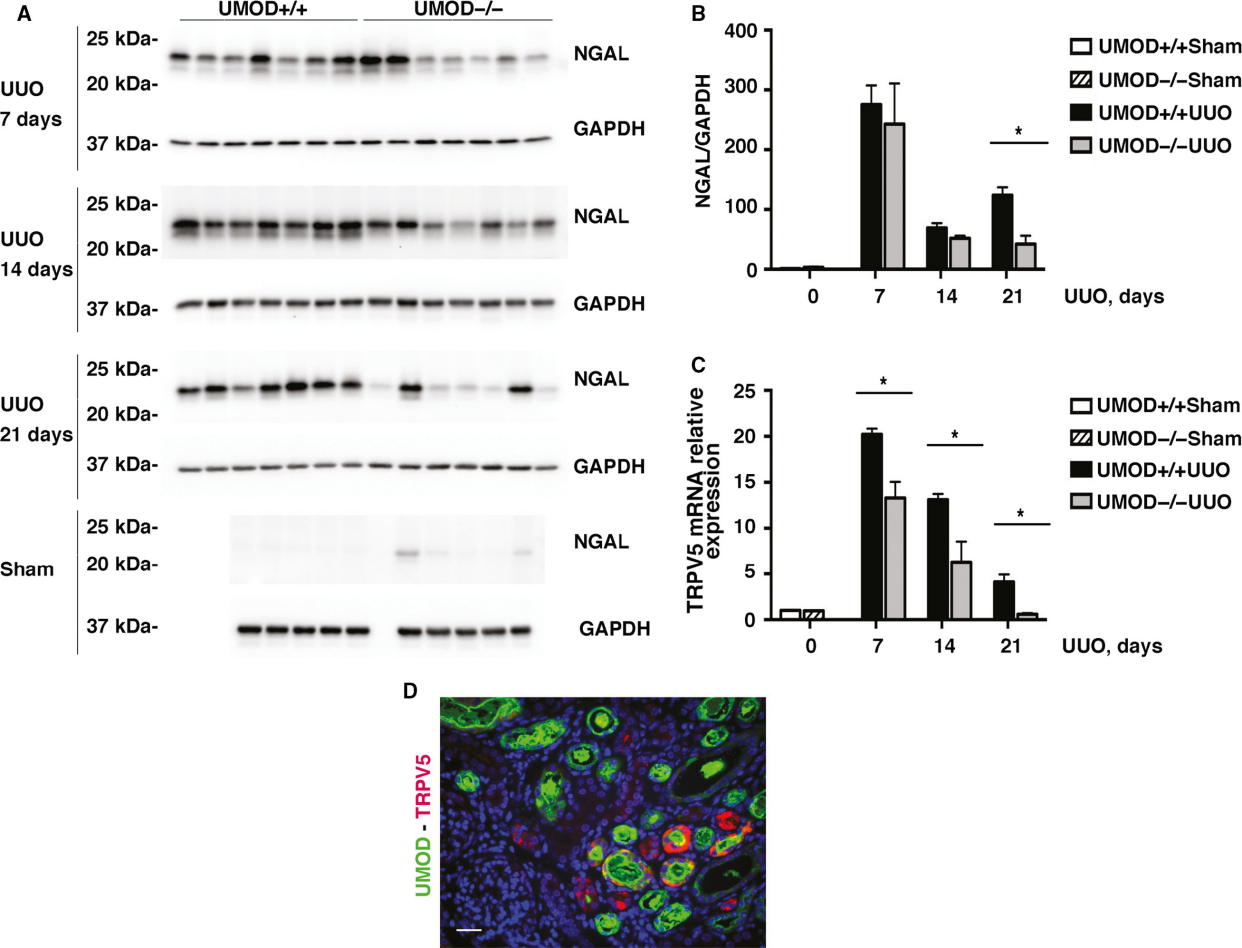
Effect of UMOD on other tubular injury markers in UUO nephropathy. Kidney NGAL protein levels, measured by western blotting, were elevated after UUO (*n* = 5 for sham groups; *n* = 7 for UUO groups) at all time‐points (days 7, 14, and 21); differences between the genotypes were only found on day 21, when they were significantly higher in the UMOD+/+ group (A, B). Kidney TRPV5 mRNA levels are markedly upregulated after UUO (*n* = 3 for sham groups; *n* = 5 for UUO groups); at all time‐points (days 7, 14, and 21) mRNA levels were significantly higher in the UMOD+/+ groups (C). By dual staining fluorescence microscopy, TRPV5 (red) was almost exclusively found in tubules with intraluminal UMOD deposits (green), as shown for a day 14 UUO UMOD+/+ kidney (D). Bar graphs represent means +1SEM; * indicates *P* value < 0.05. Photomicrograph magnification: ×40. Scale bar: 25 μm.

Neutrophil gelatinase‐associated lipocalin (NGAL) is an early and sensitive marker of injury that is selectively expressed in the TALH and collecting ducts during obstruction‐induced renal injury (Lucarelli et al. 2014). NGAL kidney protein levels were increased 7, 14, and 21 days after UUO (Fig. 4A and B), and levels were significantly lower (by 66%) on day 21 in the UMOD−/− group compared to the UMOD+/+ group. To further explore the possibility that differences in tubular injury might be selectively altered in UMOD‐expressing tubular epithelia, three transporters previously reported to interact with and/or be regulated by UMOD were investigated. Renal *ROMK2* and *NKCC2* mRNA levels were all significantly decreased compared to sham kidneys after UUO (data not shown), while *TRPV5* mRNA levels were significantly increased on days 7, 14, and 21. Only *TRPV5* levels differed significantly by UMOD genotype (1.5–7.0x lower in the UMOD−/− group; Fig. 4C). By day 21, *TRPV5* mRNA levels in the UMOD−/− mice had declined to the levels observed in the sham groups. ROMK2 and NKCC2 protein levels were too low after UUO to be meaningfully quantified by Western blotting. Differences in TRPV5 protein levels were confirmed by immunofluorescence microscopy and in the UMOD+/+ mice TRPV5 was typically expressed by tubules containing intraluminal UMOD deposits (Fig. 4D).

**Figure 5:**
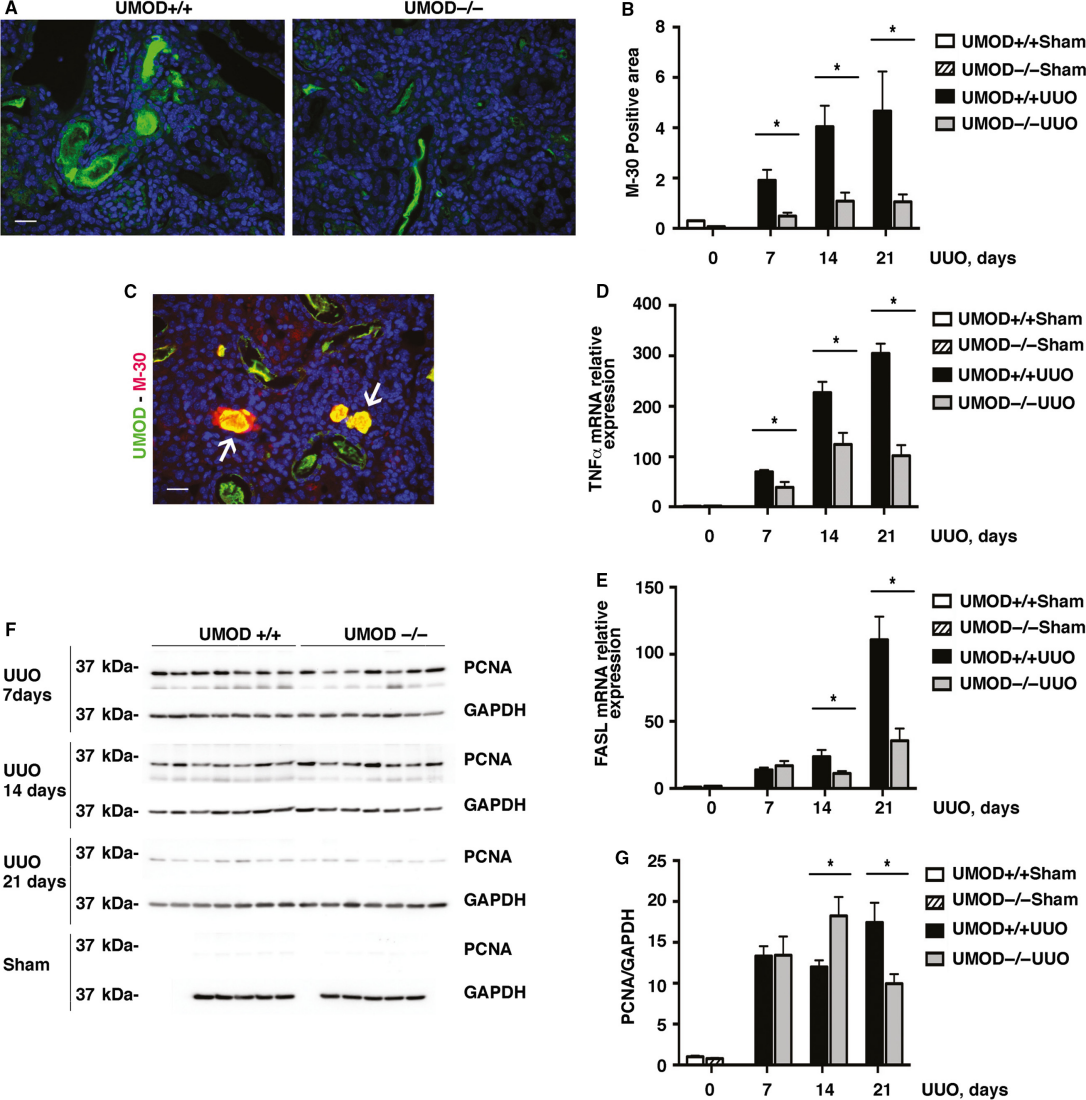
UMOD effects on apoptosis and proliferation in UUO nephropathy. Expression of M30 protein, measured by quantitative fluorescence microscopy, was used to assess the extent of apoptosis. M30 is an apoptosis‐specific neo‐antigen of epithelial cell specific cytokeratin 18. Representative day 14 photomicrographs illustrate more extensive staining in the UMOD+/+ mice (A), that is summarized quantitatively in the bar graph (B) (*n* = 3 for sham groups; *n* = 4 from UUO groups). A dual stained immunofluorescence photomicrograph illustrates intraluminal areas that express both UMOD (green) and M30 (red) (arrows; C). Kidney mRNA levels for the proapoptotic genes TNF *α* (D) and FasL (E) were significantly higher in the UMOD+/+ groups except for FasL on day 7 UUO (*n* = 3 for sham groups; *n* = 5 for UUO groups). Cell proliferation evaluated by PCNA western blotting showed a variable pattern with time after UUO; no difference on day 7, significantly higher in the UMOD−/− mice on day 14 and significantly higher in the UMOD+/+ mice on day 21 (F, G) (*n* = 5 for sham groups; *n* = 7 for UUO groups). Bar graphs represent means +1SEM; * indicates *P* value < 0.05. Photomicrograph magnification: ×40. Scale bars: 25 μm.

The early response to obstruction‐induced kidney injury is characterized by tubular epithelial cell proliferation and apoptosis. To evaluate the extent of tubular epithelial cell apoptosis, levels of M30 were quantified using immunofluorescence microscopy. M30 is an apoptosis‐specific neo‐epitope that is generated when epithelial cell‐specific cytokeratin 18 is cleaved by caspase activity during the early phase of apoptosis (Duan et al. 2003; Djudjaj et al. 2016). Both the area (Fig. 5A and B) and intensity (data not shown) of M30 immunostaining were significantly lower in the UMOD−/− mice at 7, 14, and 21 days post‐UUO surgery. At the later time‐points, a remarkable amount of the M30 protein occupied the luminal space in the UMOD+/+ mice, and colocalized with UMOD protein (Fig. 5C). Kidney mRNA levels of proapoptotic gene tumor necrosis factor alpha (*TNFα*) were significantly lower in the UMOD−/− mice (days 7, 14, and 21, Fig. 5D) while proapoptotic FAS ligand (*FASL*, a member of the TNF family) mRNA levels were significantly lower on days 14 and 21 (Fig. 5E). Kidney proliferating cell nuclear antigen (PCNA) kidney protein levels increased more than 10‐fold after UUO and showed significant time‐course‐dependent differences between the genotypes. PCNA levels were significantly higher on day 14 and lower on day 21 after UUO in the UMOD−/− groups (Fig. 5F).

Since UMOD is known to be expressed in renal primary cilia, and mutant human *UMOD* genes have been reported to decrease ciliary UMOD expression (Zaucke et al. 2010), levels of acetylated *α*‐tubulin were measured by immunoblotting to determine if the absence of UMOD during UUO‐induced kidney injury altered the expression of renal primary cilia. Acetylated *α*‐tubulin levels in UUO kidneys were significantly lower than sham levels (Days 7, 14), with no differences detected between the UMOD+/+ and −/− groups (data not shown).

Evidence for calcium or phosphate deposition was investigated by alizarin red and Von Kossa staining, respectively, as (1) renal papillary calcification and stone formation have been reported in older UMOD−/− mice (Liu et al. 2010), (2) interstitial phosphate deposition has been implicated as a contributing pathogenic factor in renal fibrosis, and (3) TRPV5 is a gatekeeper protein for active renal calcium reabsorption in the kidney (Oddsson et al. 2015; Nie et al. 2016; Shen et al. 2016). With the use of positive controls to confirm the staining protocols, all of the sham and UUO kidneys evaluated showed no positive tubulointerstitial reactivity with either alizarin red or Von Kossa (data not shown).

Noting that UMOD‐deficiency has been reported to render mice more susceptible to bladder colonization with *Escherichia coli*, it was important to ensure that none of the experimental mice had evidence of infection. Using a gram staining protocol optimized for use in tissue sections, reactive bacteria were not detected in any of the kidney tissues examined (data not shown).

### Interstitial inflammation

**Figure 6:**
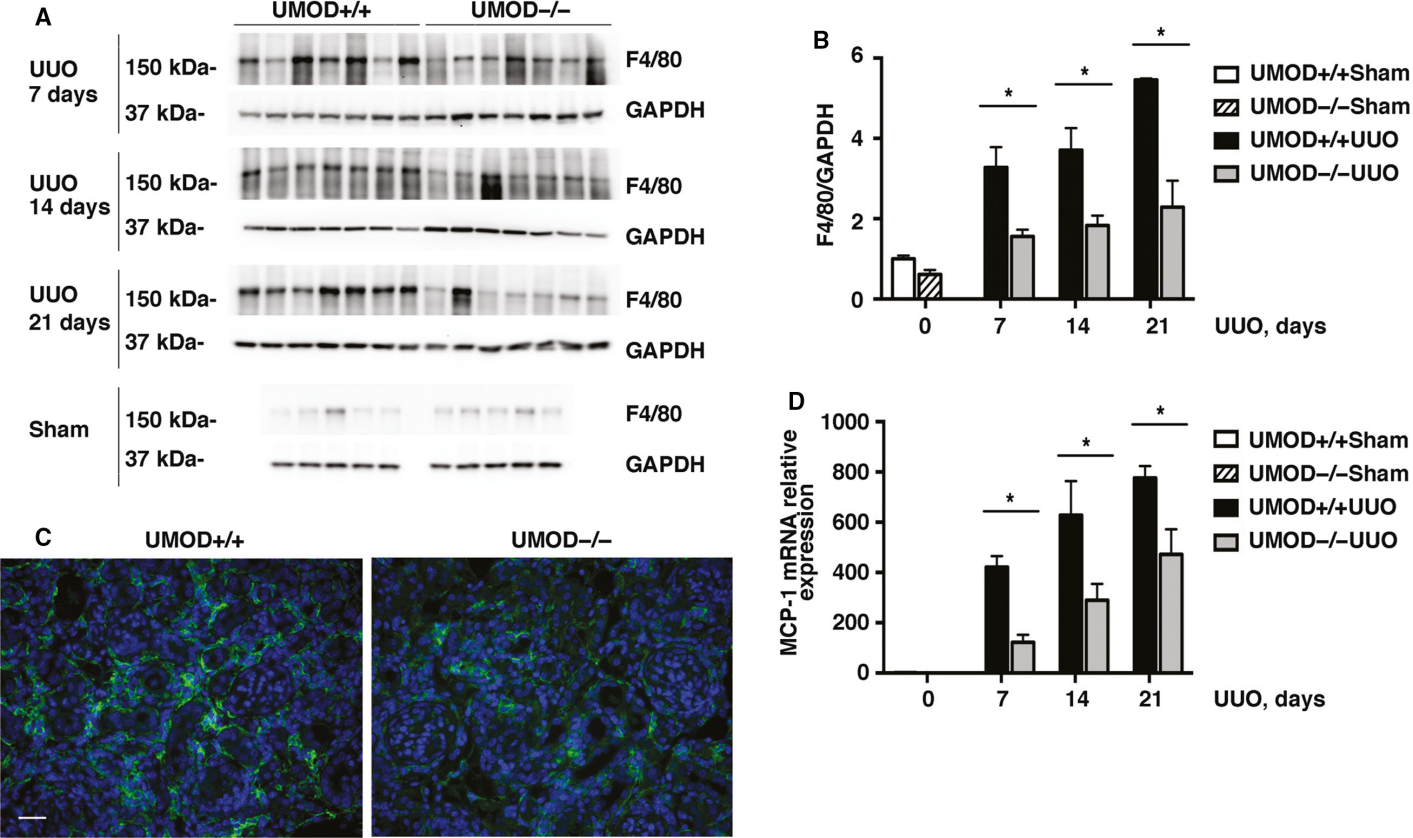
UMOD expression amplifies UUO‐associated interstitial inflammation. Interstitial inflammation is a hallmark feature of kidney fibrosis. Interstitial macrophage density, quantified by kidney F4/80 protein western blotting (*n* = 5 for sham groups; *n* = 7 for UUO groups), was significantly lower in the UMOD−/− groups by 41–58% at all time‐points (A, B). Representative day 21 immunofluorescence photomicrographs illustrate a smaller interstitial area expressing the F4/80 macrophage protein (green) in the UMOD−/− kidney (C). Kidney MCP‐1 mRNA levels were significantly lower in the UMOD−/− mice on UUO days 7, 14, and 21 (*n* = 3 for sham groups; *n* = 7 for UUO groups) (D). Bar graphs represent means +1SEM; * indicates *P* value < 0.05. Photomicrograph magnification: ×40. Scale bar: 25 μm.

Interstitial macrophage infiltration is a well‐established feature of the pathological responses that culminate in kidney fibrosis (Ricardo et al. 2008). Though our study did not detect immunohistochemical evidence of UMOD protein deposits within the interstitium after UUO‐induced injury, other mechanisms have been proposed that suggest a potential link between UMOD and inflammation. The mouse intracellular glycoprotein F4/80 is expressed by most tissue macrophages. By western blotting, F4/80 protein levels increased after UUO, as previously reported in several studies, but the severity differed between the UMOD+/+ and UMOD−/− groups. F4/80 protein levels were significantly higher in the UMOD+/+ mice on days 7, 14, and 21 after UUO (Fig. 6A and B). Kidney mRNA levels for monocyte chemoattractant protein‐1 (MCP‐1) (Fig. 6C) and RANTES (data not shown) were remarkably increased after UUO; levels were significantly lower in the UMOD−/− group compared to the UMOD+/+ group at all time‐points.

### Kidney fibrosis

**Figure 7:**
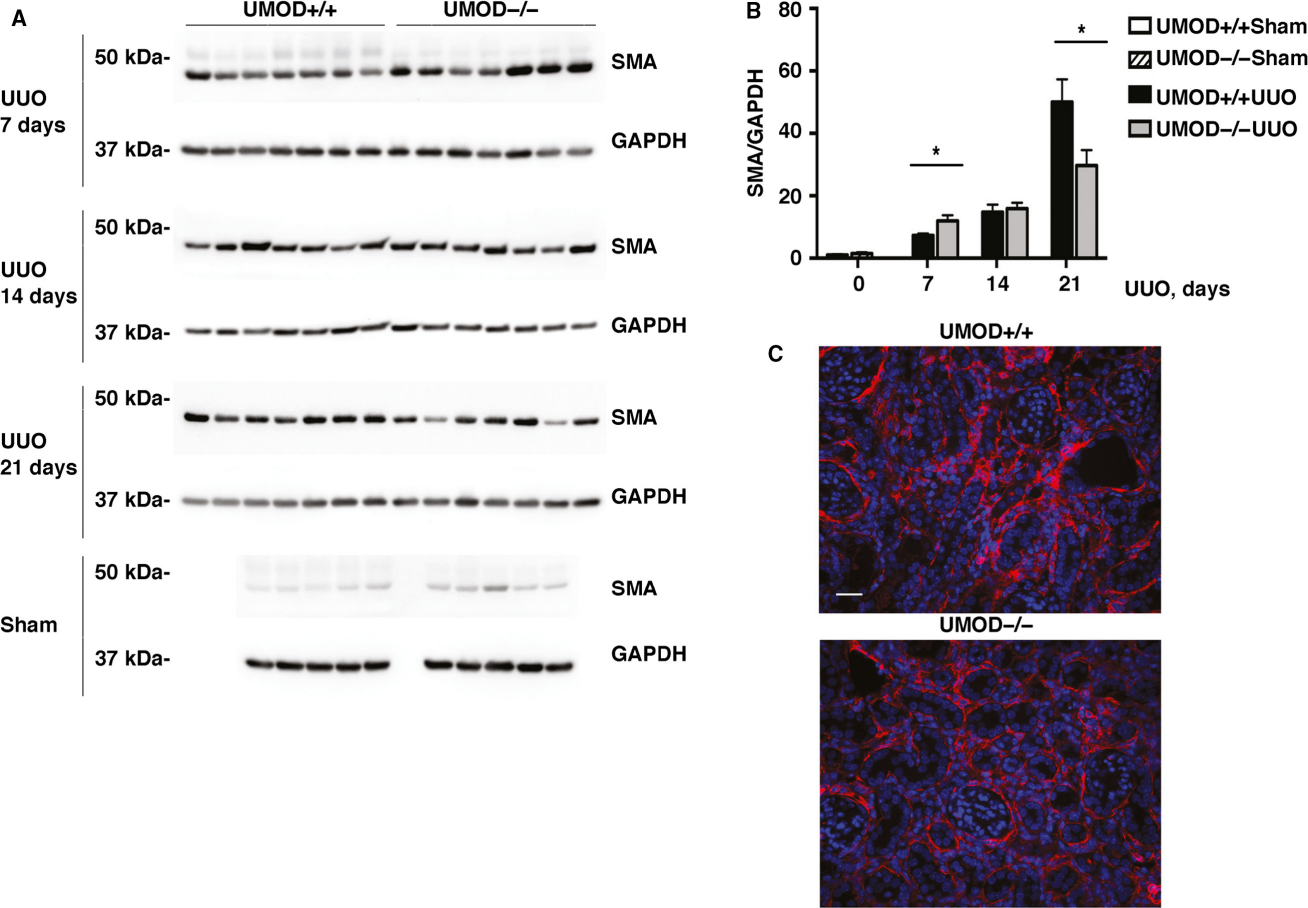
UMOD effects on interstitial myofibroblasts in UUO nephropathy. The intensity of the interstitial myofibroblast infiltration, evaluated by *α* SMA western immunoblotting (*n* = 5 for sham groups; *n* = 7 for UUO groups), revealed differences in the kinetics of the response, with significantly lower levels on day 7 and higher levels on day 21 in the UMOD +/+ mice (A, B). Representative immunofluorescence photomicrographs illustrate the differences in *α* SMA staining (red) on day 21, with DAPI nuclear staining in blue (C). Bar graphs represent means +1SEM; * indicates *P* value < 0.05. Photomicrograph magnification: ×40. Scale bar: 25 μm.

**Figure 8:**
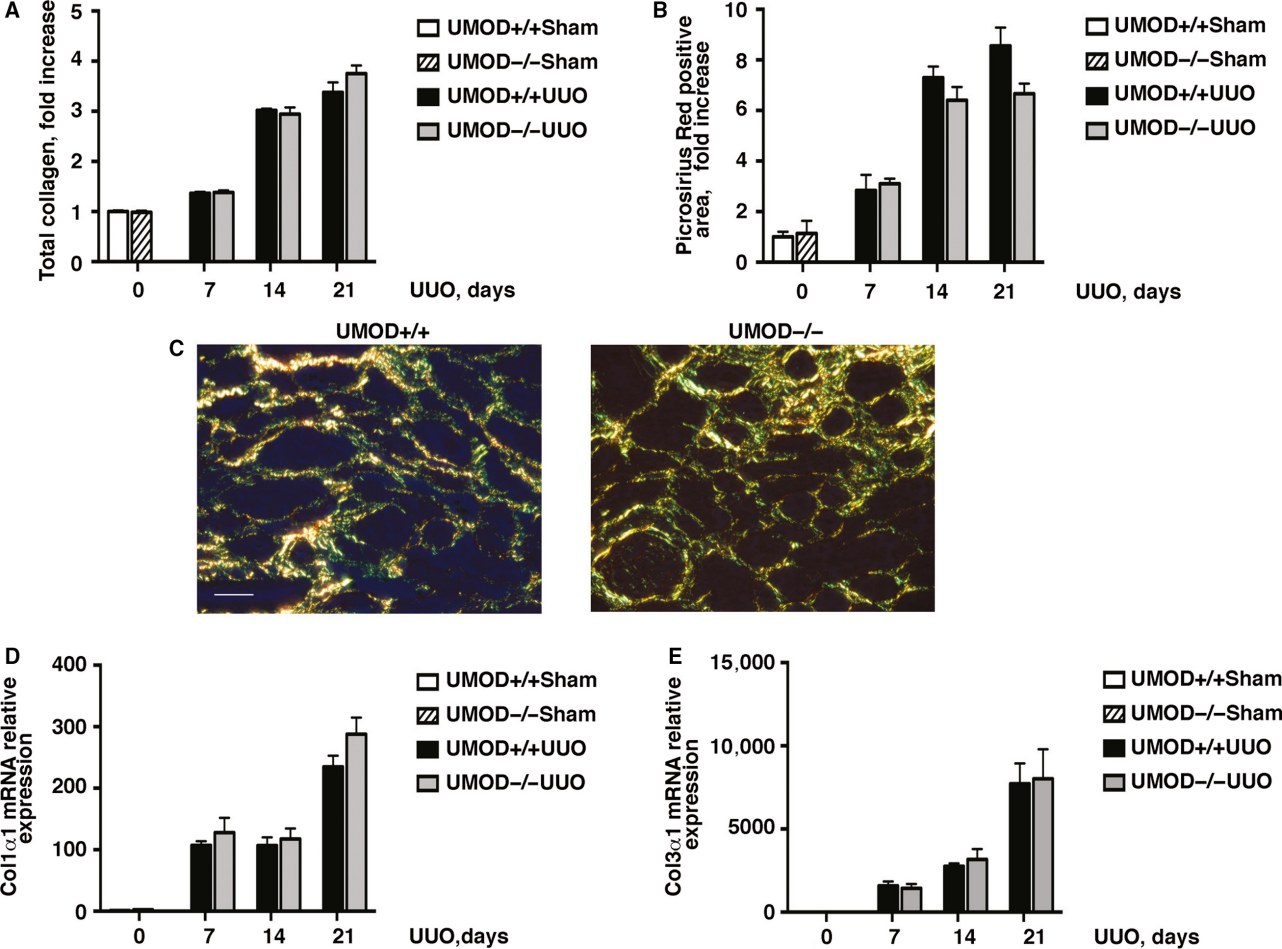
UMOD expression does not alter kidney fibrosis severity in UUO nephropathy. Total kidney collagen levels (*μ*g/mg wet kidney weight) calculated using the hydroxyproline assay and presented as fold increase relative to sham values (A) and picrosirius red+ interstitial areas (B) were similar between the UMOD+/+ and UMOD−/− groups at all time‐points after UUO (*n* = 10 per group and *n* = 3–4 per group for the respective methods). Representative polarized light microscopic photomicrographs from day 21 kidney sections illustrate a similar extent of picrosirius red+ interstitial collagen deposits (yellow) (C). Kidney mRNA levels measured by real‐time PCR (*n* = 3 for sham groups; *n* = 5 for UUO groups) were similar for procollagen 1*α*1 (D) and procollagen 3*α*1 (E). Bar graphs represent means +1SEM. Photomicrograph magnification: ×40. Scale bar: 25 μm.

The severity of kidney fibrosis is extensively used in both animal and human studies as a reliable predictor of long‐term kidney outcome. Most studies use collagen as the key biomarker, though it is still unknown if collagen or another of the many extracellular matrix proteins that accumulate in chronically damaged kidneys is the best biomarker (Eddy et al. 2012). The majority of the scar‐forming matrix is produced by interstitial myofibroblasts, identified in tissues as cells expressing alpha smooth muscle actin (*α*SMA). As expected, interstitial myofibroblast density (measured by *α*SMA immunoblotting) was increased after UUO, but the kinetics of this response differed between the genotypes; *α*SMA levels were significantly higher on day 7, not different on day 14 and lower on day 21 in the UMOD−/− groups (Fig. 7A–C). Total kidney collagen levels (expressed as *μ*g collagen/mg wet kidney weight) were increased and similar between the UMOD+/+ and UMOD−/− groups at all time‐points after UUO (Fig. 8A). The lack of a difference in kidney collagen levels between UMOD +/+ and UMOD−/− groups was confirmed by picrosirius red staining (Fig. 8B and C). Kidney mRNA levels for procollagen1*α*1 and procollagen3*α*1 were dramatically increased at all times after UUO relative to sham kidney levels, but there were no significant differences between the *Umod* genotypes (Fig. 8D and E). Given that UMOD has a reported affinity for fibronectin (FN), kidney FN levels were measured by western blotting and were also found to be increased but similar between the UMOD+/+ and UMOD−/− groups at all time‐points after UUO, with the single exception that levels were significantly lower in the UMOD−/− group on day 21 (data not shown).

### UMOD and KIM‐1 in vitro Studies

**Figure 9:**
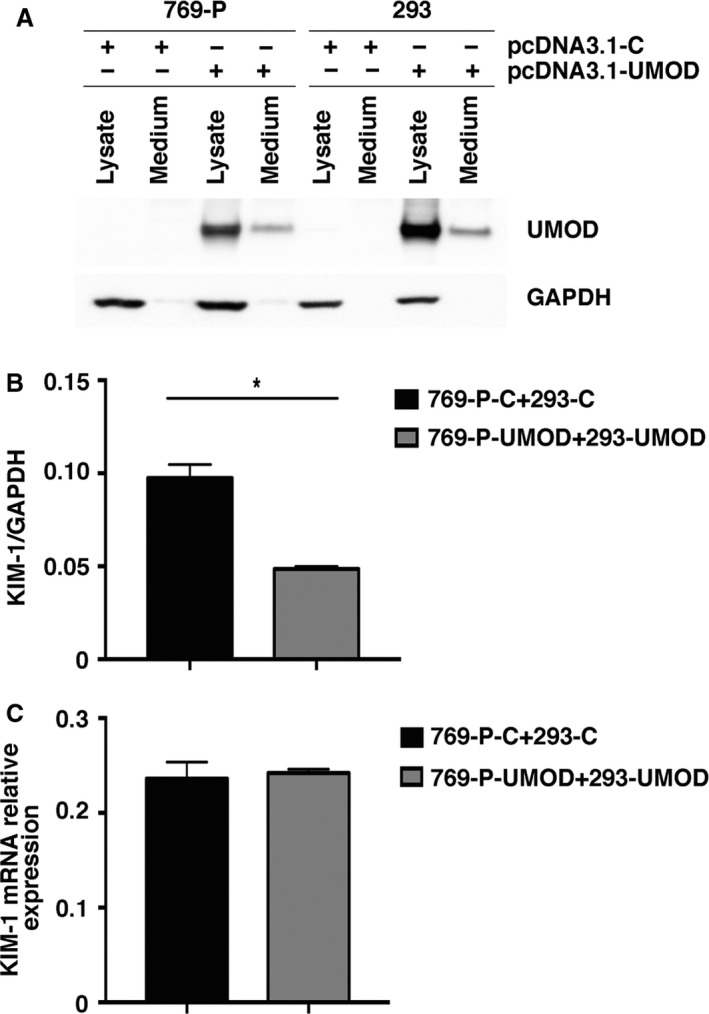
UMOD reduces tubular cell KIM‐1 levels in vitro. Preliminary studies to maximize the exposure of KIM‐1 producing 769‐P cells to UMOD demonstrate that both 769‐P and 293 cells transiently transfected with a pcDNA3.1 vector expressing human *UMOD* (pcDNA3.1‐UMOD) produced readily detectable UMOD protein levels within cells and their conditioned medium compared with cells transfected with a pcDNA3.1 vector expressing a noncoding control vector (pcDNA3.1‐C) (A). Under transwell culture conditions, 769‐P cells transfected with pcDNA3.1‐UMOD (769‐P‐UMOD) and harvested from the upper chamber after exposure to UMOD from both the upper and lower chambers for 48 h, expressed significantly lower KIM‐1 protein levels compared with cells transfected with pcDNA3.1‐C (769‐P‐C) and not exposed to UMOD (B). Lower chambers contained 293 cells transfected with pcDNA3.1‐C (293‐C) or pcDNA3.1‐UMOD (293‐UMOD). Results are western blot data corrected for GAPDH protein levels (*n* = 3). KIM‐1 mRNA levels in 769‐P cells cultured under the same conditions (*n* = 3) were similar between control transfected and UMOD transfected cells (C). * indicates *P* value < 0.05.

Given the striking effect of UMOD deficiency on enhanced KIM‐1 expression after obstruction‐induced kidney, in vitro studies were performed to determine if UMOD can down‐regulate KIM‐1 expression in a human cell line derived from a renal adenocarcinoma that is known to express KIM‐1 (769‐P) (Zhang et al. 2007). Since UMOD‐triggered renal tubular cell signaling pathways have not been identified, these experiments were limited to evaluating responses to UMOD protein. Because commercially available purified human UMOD is prohibitively expensive, we optimized transient transfection conditions using a human *UMOD* expressed in the pcDNA3.1 vector to maximize the amount of UMOD secreted into the culture media. After multiple culture optimization experiments, a transwell design was selected, placing human embryonic kidney 293 cells in the lower chamber as the primary source of UMOD, with the responder 769‐P cells in the upper chamber. Experiments were conducted with both cells line transfected with control (C) or *UMOD* DNA. Transfection efficiency was confirmed by UMOD immunoblotting. The highest level of UMOD expression was achieved at 48 h after transfection, and at this point the protein was detected in samples harvested from both the cells and their conditioned media (Fig. 9A). Compared to control cells (769‐P‐C) co‐cultured with 293‐C, 769‐P‐UMOD cells exposed to UMOD in medium from 293‐UMOD cells demonstrated significantly reduced KIM‐1 protein level (Fig. 9B) while mRNA levels were similar (Fig. 9C).

## Discussion

UMOD is an abundant kidney‐specific protein that has garnered renewed attention in recent years due to growing evidence of important linkages with human chronic kidney disease. Unlike the compelling evidence that mutant UMOD protein, caused by autosomal dominant inheritance of a mutant gene, mediates chronic tubulointerstitial damage, definitive evidence that genetically normal human UMOD directly contributes to either protection against or propagation of chronic kidney disease is still lacking.

Seeking to answer this question, we reasoned that the obstruction model of chronic kidney disease would be ideal to track the production and fate of UMOD during sustained kidney injury and to pursue potential pathogenic mechanisms in vivo by comparing outcomes in mice that were genetically identical other than lacking a functional *Umod* gene. In the UMOD+/+ groups, UMOD protein expression was 9–13x increased above sham levels despite nonsignificant changes in kidney mRNA levels indicating intrarenal protein retention. In the face of obstruction this protein, that is normally shed and excreted into the urine, was indeed trapped. What was remarkable was the appearance of highly organized intraluminal aggregates, consistent with its disulfide bond‐rich tertiary structure and its tendency to polymerize. These cast‐like formations were not limited to the site of UMOD secretion, but could also be detected within some proximal tubules. At the cellular level, UMOD moved from its traditional predominantly apical location to a more intracellular location.

The most significant findings in this study are the extent to which tubular injury and interstitial inflammation were modified when UMOD was absent. Significantly lower levels of the epithelial cell apoptotic marker M30 in the UMOD−/− mice indicate that the number of apoptotic cells and cellular debris were reduced. While differences in apoptotic rates likely contributed given higher levels of the proapoptotic genes TNF*α* and FasL in the UMOD+/+ groups, delayed apoptotic cell clearance was likely an important contributing factor. Large intraluminal M30 positive cellular debris was frequently observed on days 14 and 21. In the wild‐type mice, dual staining with UMOD was observed suggesting that dead cells became trapped within UMOD gel‐like networks and were unable to move further down the nephron. One of the most remarkable findings in this study was the striking up‐regulation of KIM‐1, to levels that were 4–13x higher in the UMOD−/− mice. Deciphering the primary function of KIM‐1, that is expressed de novo by injured proximal tubules, is still under active investigation but it is known to function as a scavenger receptor to help clear luminal debris and aid in tissue repair and regeneration (Yang et al. 2015). KIM‐1 recognizes phosphatidylserine residues on the outer surface of luminal apoptotic cells and functions as an endocytic scavenger receptor to eliminate them. Increased intraluminal M30 positive apoptotic epithelia in the wild‐type mice raises the possibility that secreted UMOD glycoprotein, by trapping KIM‐1 or blocking its mucin domain so that it is not able to interact with specific epitopes on apoptotic debris and/or by interfering with KIM‐1‐mediated uptake of apoptotic debris by dedifferentiated tubular epithelia, impairs the efficiency of KIM‐1 apoptotic cell scavenging pathway (Yang et al. 2015; Arai et al. 2016). KIM‐1 levels were not only much higher in the UMOD−/− mice, but a larger portion was retained within tubular epithelial cells in the animal studies. The in vitro data also suggest that UMOD suppresses renal epithelial KIM‐1 levels. It is possible that KIM‐1‐rich epithelial cells in the UMOD‐deleted environment are more efficient scavengers of apoptotic debris, as KIM‐1 has been reported to induce the differentiation of injured epithelial cells to phagocytes (Ichimura et al. 2008).

The intensity of the interstitial inflammatory response, as evaluated by F4/80 monocyte/macrophage protein levels, was significantly lower in the UMOD−/− mice during the course of UUO. In contrast to findings in a few published studies (Resnick et al. 1978; Zager et al. 1978; Howie and Brewer 1983; Bachmann et al. 1985; Chambers et al. 1986; Akioka et al. 2009; El‐Achkar et al. 2013), the absence of convincing interstitial UMOD deposition in the present study suggests that the remarkable differences in the interstitial inflammation severity were likely driven by differences in tubular responses. The mRNA levels for two important chemokines involved in macrophage recruitment, MCP‐1 and RANTES, derived at least in part from proximal tubules, were significantly lower in the UMOD−/− mice and likely contributed to the less robust inflammatory response. Lower chemokine levels might reflect less severe tubular injury but this difference may also be due to higher KIM‐1 levels, as Yang et al. (2015) recently reported that KIM‐1 has an anti‐inflammatory effect due to its ability to down‐regulated NF‐*κ*B activity and inflammatory cytokine release. Remarkably, despite 50% lower F4/80 levels throughout the study, the lack of a difference in fibrosis severity suggests that the absence of UMOD results in the recruitment of interstitial macrophages that are distinct and functionally polarized to a more robust fibrogenic phenotype than those recruited when UMOD is present. Furthermore, consistent differences in the number of *α*SMA positive interstitial myofibroblasts were not seen over the 3‐week observation period, with the consequence that interstitial collagen genes procollagens I and III and interstitial collagen accumulation were unaffected by the *Umod* genotype. This finding was confirmed using two different measures of fibrosis; the hydroxyproline‐based biochemical measurement of total kidney collagen and the picrosirius red‐stained interstitial collagen area on kidney tissue sections.

Three additional observations suggest that renal tubular cells in the UMOD−/− mice were better defended after the onslaught of obstruction. First, they showed a more robust proliferative response on day 14 that waned by day 21, perhaps indicating better repair. Second, *TRPV5* mRNA levels (a gene expressed by late distal convoluted tubules and collecting tubules) had returned to baseline levels in the UMOD−/− mice by day 21 after 1.5–2.1x increases on days 7 and 14. Third, elevated kidney NGAL protein levels (reflecting injured TALH and collecting ducts) were also lower (66%) on day 21 in the UMOD−/− mice. While previous reports found that UMOD up‐regulates TRPV5 by impairing its caveolin‐mediated endocytosis (Wolf et al. 2013), *TRPV5* mRNA levels showed a sustained elevation in the UMOD+/+ mice after UUO, supporting a role for increased UMOD‐dependent gene transcription via either direct or indirect mechanisms. Though fibrosis severity is an excellent CKD outcome predictor due to its close correlation with the loss of intact functional nephron units., it is increasingly acknowledged that fibrosis is really a surrogate measure of nephron loss due to tubular atrophy. Unfortunately we currently lack reliable and easy‐to‐perform histological measures of intact and functional epithelial cells that could be used as a clinically reliable predictor of renal prognosis. One outcome difference that is not yet explained is why in a model of acute reversible kidney injury induced by ischemia‐reperfusion, renal recovery was significantly delayed in the UMOD−/− mice (El‐Achkar et al. 2013). In that study, differences in the inflammatory response were observed and may have been related to unique pattern of uromodulin distribution during the recovery phase – along the basolateral domain of TALH cells and within the interstitium, which was not the pattern observed in this UUO study (El‐Achkar et al. 2013).

Evidence is emerging from studies in humans and experimental mice to suggest that UMOD has pleiotropic and context‐dependent effects. Humans expressing CKD risk‐associated polymorphic variants in the UMOD gene promoter and UMOD‐overexpressing transgenic mice both develop foci of renal damage characterized by tubular casts, tubular dilatation and detached tubular epithelial cells (Trudu et al. 2013), but these changes alone do not appear to cause CKD. However, in the face of primary renal injury, such as obstructive nephropathy in the present study, the expression and location of UMOD appears to determine if it will have detrimental effects on disease severity. In this context, UMOD is known to promote inflammation but its effect on delaying the clearance of apoptotic cells has not been reported previously.

This study confirms previous cautionary observations about the measurement of urinary UMOD levels as a potential CKD biomarker. Recent studies have suggested that urinary levels collected outside the context of acute kidney injury are a useful biomarker of tubular reserve, UMOD genotype and renal prognosis (Han et al. 2013; Trudu et al. 2013; Zhou et al. 2013; Devuyst and Bochud 2015; Garimella et al. 2015; Pruijm et al. 2016; Troyanov et al. 2016). However, these assays impose significant technical challenges related to sample handling, as this disulfide‐bond‐rich glycoprotein readily self‐aggregates and forms precipitates (Youhanna et al. 2014), casting doubt on its practical utility as a biomarker. Even when a group of day 7 UUO mice were specifically selected for these urinary studies and the samples were rapidly collected and processed, it was evident that a significant fraction of the protein had precipitated. As methods are refined for future human studies, UMOD levels in both the soluble form and the resolubilized UMOD‐containing precipitated fraction will need to be measured and corrected to the urinary creatinine concentration to obtain reliable, reproducible and meaningful data.

A limitation of this study based on the UUO model of sustained kidney injury is the inability to include a measure of kidney function as one of the study outcomes. To do so will require studies in another experimental chronic kidney disease model, which are currently in progress in our laboratory using the folic acid nephropathy model.

In summary, this study based on the UUO model, demonstrated that kidney UMOD protein levels increase within both the cytoplasm of TALH cells and tubular luminal spaces over the 21 day observation period and contribute to disease severity. In comparison to mice with genetic UMOD deficiency, tubular injury and apoptosis are more severe and associated with a more intense inflammatory response. Though fibrosis severity was similar between the time‐matched UMOD+/+ and UMOD−/− groups, worse tubular damage associated with UMOD activity is likely the more relevant kidney outcome measure. Lower KIM‐1 levels associated with impaired apoptotic cell scavenging and proinflammatory activities, appear to be UMOD‐regulated pathways during the progression of chronic kidney injury.

## Conflict of Interest

None declared.
